# Cerebrolysin Ameliorates Age-Induced Dendritic Spine Degeneration and Memory Decline in C57BL6 Mice

**DOI:** 10.1007/s11064-025-04627-0

**Published:** 2025-12-29

**Authors:** Leonardo Aguilar-Hernández, Gabriel Daniel Flores-Gómez, Juan Nacher, Julio Cesar Morales-Medina, Gonzalo Flores

**Affiliations:** 1 Instituto de Fisiología, Benemérita Universidad Autónoma de Puebla (BUAP), 14 Sur 6301, Puebla, 72570 Mexico; 2 Centro de Investigación en Reproducción Animal, Cinvestav-UAT, Tlaxcala, 90120 Mexico; 3 Neurobiology Unit, Institute for Biotechnology and Biomedicine (BIOTECMED), Universitat de Valencia, Valencia, 46100 Spain; 4 Spanish National Network for Research in Mental Health CIBERSAM, Madrid, 28029 Spain; 5 Fundación Investigación Hospital Clínico de Valencia, INCLIVA, Valencia, 46010 Spain

**Keywords:** Aging, Cerebrolysin, Dendritic spines, Hippocampus, Prefrontal cortex, Recognition memory

## Abstract

Aging is associated with progressive synaptic deterioration and cognitive decline; however, therapeutic strategies capable of restoring both structural and functional deficits remain limited. This study evaluated the effects of aging on dendritic spine dynamics and recognition memory across multiple brain regions, and evaluated whether chronic treatment with cerebrolysin (CBL) could ameliorate age-related alterations (3, 6, 12 and 18 months of age). We additionally assessed the effects of CBL on key molecular markers of synaptic plasticity in aged (18-month) C57BL6 mice. Aging impaired locomotor activity (12- and 18-month groups) and produced deficits in short- and long-term recognition memory relative to young controls. Notably, CBL selectively enhanced locomotion in 18-month group and improved short-term memory in the 12-month group. At the structural level, aging reduced spine density and decreased the proportion of thin and mushroom spines in the prefrontal cortex and dorsal hippocampus, whereas CBL treatment increased spine density in the dorsal hippocampus and basolateral amygdala, and promoted the formation of mature mushroom spines in a region and age-dependent manner. Importantly, CBL elevated β-actin, synaptophysin and brain-derived nerve factor expression across multiple regions in the 18-month group. This study provides the first integrated demonstration that CBL enhances dendritic spine maturation and dendritic structural remodeling while concurrently improving cognitive outcomes within the same cohort of aged animals. Collectively, our findings position CBL as a promising therapeutic candidate to counteract age-related synaptic loss and cognitive decline, advancing current understanding of neuroprotective interventions in aging.

## Introduction

Aging is a natural process in living beings characterized by the progressive decline of the physiology and anatomy of their tissues. The impact of aging on the central nervous system (CNS) generates a decline in cognitive functions [1], which causes an increase in the prevalence of dementia and mild-cognitive impairments [2]. These declines appear to be the consequence of neuronal damage and decreased efficiency of maintenance and repair systems, which is exacerbated in key regions that govern cognitive processes by having higher metabolic rates [3].

Structures of the limbic system such as the hippocampus and the amygdala, and their connections with cortical regions such as the entorhinal and prefrontal cortex (PFC) are essential for the execution of cognitive processes such as memory, motivation, and spatial processing [4, 5]. The function of these communication pathways depends on their high plasticity to modify their neural networks. This intercommunication and plasticity occur between the neurons of each region mainly through excitatory synapses on dendritic spines [6]. The density, morphology and function of dendritic spines are correlated with factors such as the stimulation they receive, their intrinsic capacity to form and maintain these structures, the contribution of neurotrophic factors, oxidative stress, and glial function, in addition to other mediators. Dysfunctions in plastic processes impact the morphology and number of dendritic spines, which can be reflected as alterations in cognitive processes [7, 8].

Throughout the course of brain aging, intracellular and extracellular environments such as oxidative stress, cell damage, and inflammation reduce their neuroplastic capacity. The expression of proteins relevant to neuronal plasticity such as post-synaptic density protein 95 (PSD95), brain-derived neurotrophic factor (BDNF) and nerve growth factor (NGF) are decreased [9] along with antioxidant defenses and neurotrophic factors [10], while certain proinflammatory cytokines are increased [11]. In addition, an increase in astrocytes and reactive microglia is observed, as well as a reduction in the rate of neurogenesis [9, 12]. At the neuronal level, reductions in dendritic length, the density of dendritic spines and modifications in the proportion of mushroom dendritic spines to inefficient stubby dendritic spines have been found in limbic cortical regions [13, 14]. Taken together, these changes produce alterations in long-term potentiation [15], reflected as declines in processing speed, working memory and executive function [16], affectations in spatial memory [17], locomotor activity and exploratory behavior [18].

Multiple studies have shown that the administration of substances with anti-inflammatory and antioxidant properties can reduce morphological, functional, and behavioral declines, in addition to increasing the presence of neurotrophic factors in aging [10, 19–23]. In this regard, cerebrolysin (CBL) is a complex of protein derivatives obtained from the enzymatic degradation of porcine brain tissue. Its components have the ability of crossing the blood-brain barrier, which allows peripheral administration. CBL’s effects on the brain resemble the administration of endogenous neurotrophic factors such as BNDF, since it exerts pleotropic effects of neuroprotection and neuroregeneration [24]. CBL has the potential to reduce the negative effects of brain aging and neurodegenerative diseases by exerting neurotrophic actions [25, 26]. The underlying mechanisms include processes such as regulation of receptors and intracellular pathways, reduction of the production of AB and TAU-hyperphosphorylation; and increases in neurotrophic factors, neuroplasticity and neurogenesis [27], among others.

The selection of animal species and model in this study directly align with the scientific objectives, allowing investigation of age-related brain modifications and the potential neuroprotective effects of CBL. Our group has previously shown that CBL mitigates neuronal and behavioral alterations in animal models of schizophrenia [28], autism [29], vascular dementia [30, 31] and metabolic syndrome [32]. Additionally, other studies have shown that CBL administration decreases the degree of oxidative stress and apoptosis in the brain and improves memory, learning and locomotor activity in models of aging [33] and Parkinson’s disease [34]. Clinically, CBL provides neuroprotection in patients recovering from stroke [35–37] and traumatic brain injury [38], supporting translational relevance. In the present study, we examine the effects of CBL treatment in rodents of different ages (3, 6, 12 and 18 months of age) to assess and compare neurobiological and behavioral modifications associated with aging. Moreover, in the 18-month group, we assessed the effects of CBL on key molecular markers of synaptic plasticity [synaptophysin (Syn), α-synuclein, neuronal nitric oxide synthase (nNOS), β-actin and BDNF].

## Material and Method

### Animals

3-, 6-, 12- and 18-month-old C57BL6 male mice were acquired from the Claude Bernard animal facility of the Benemérita Universidad Autónoma de Puebla. All breeding and maintenance were conducted by the institutional animal facility. No previous procedures were conducted in mice used in the present study. Animals were kept under constant humidity and temperature conditions with a 12-h light-dark cycle with food and water *ad libitum*. The experimental unit is a single animal. Each age group was subdivided into 2 subgroups: CBL (2 ml/kg; Ever Neuro Pharma GmbH, Unterach, Austria) or vehicle solution in the same volume (Hartmann solution, PiSA Pharmaceutics, México). The treatment was applied 5 days/week for 2 months. The sample size was calculated based on prior studies from our group using similar tests. The selection of animals was randomized according to a pre-generated random number table. All procedures were carried out in accordance with the regulations of the BUAP Animal Care Committee (FLAG-UALVIEP-17-1), the guide for the use and care of laboratory animals of the Mexican Consulate for animal care (NOM-062-ZOO-1999) and ARRIVE guidelines [39]. Animals were excluded from the study if they exhibited any signs of distress, particularly the 18-month-old mice with humane endpoints established in the animal facility. All animals that reached the designated experimental age were included in the study.

### Behavior Tests

The behavioral tests were carried out between 9:00 a.m. to 12:00 p.m. in a noise-insulated and odor-free room with red light. Animals were kept in this room 24 h before starting the tests for their conditioning. All tests conducted were videotaped (Steren HD webcam, 1080p, 15/30 fps) for later analysis. The novel object recognition (NOR) test was conducted following the same sequence as in previous sessions to minimize potential confounding factors. Animal numbers for the behavioral test were as follows: 3-months-old (*n* = 8), 6-months-old (*n* = 7), 12-months-old (*n* = 7), and 18-months-old (*n* = 3) per group.

#### Locomotor Activity Assessment

To measure locomotor activity, a 40 cm^2^ wooden box was used, which had its floor subdivided into sixteen 4 × 4 quadrants of 10 cm^2^ [8]. At the beginning of the test, the rodent was placed in the center of the box and was allowed to roam freely. For the analysis of locomotor activity, the number of quadrants traversed for 10 min was quantified, obtaining an estimate of the distance traveled in centimeters. The box was washed and disinfected before introducing a new rodent.

#### Recognition Memory

**Fig. 1:**
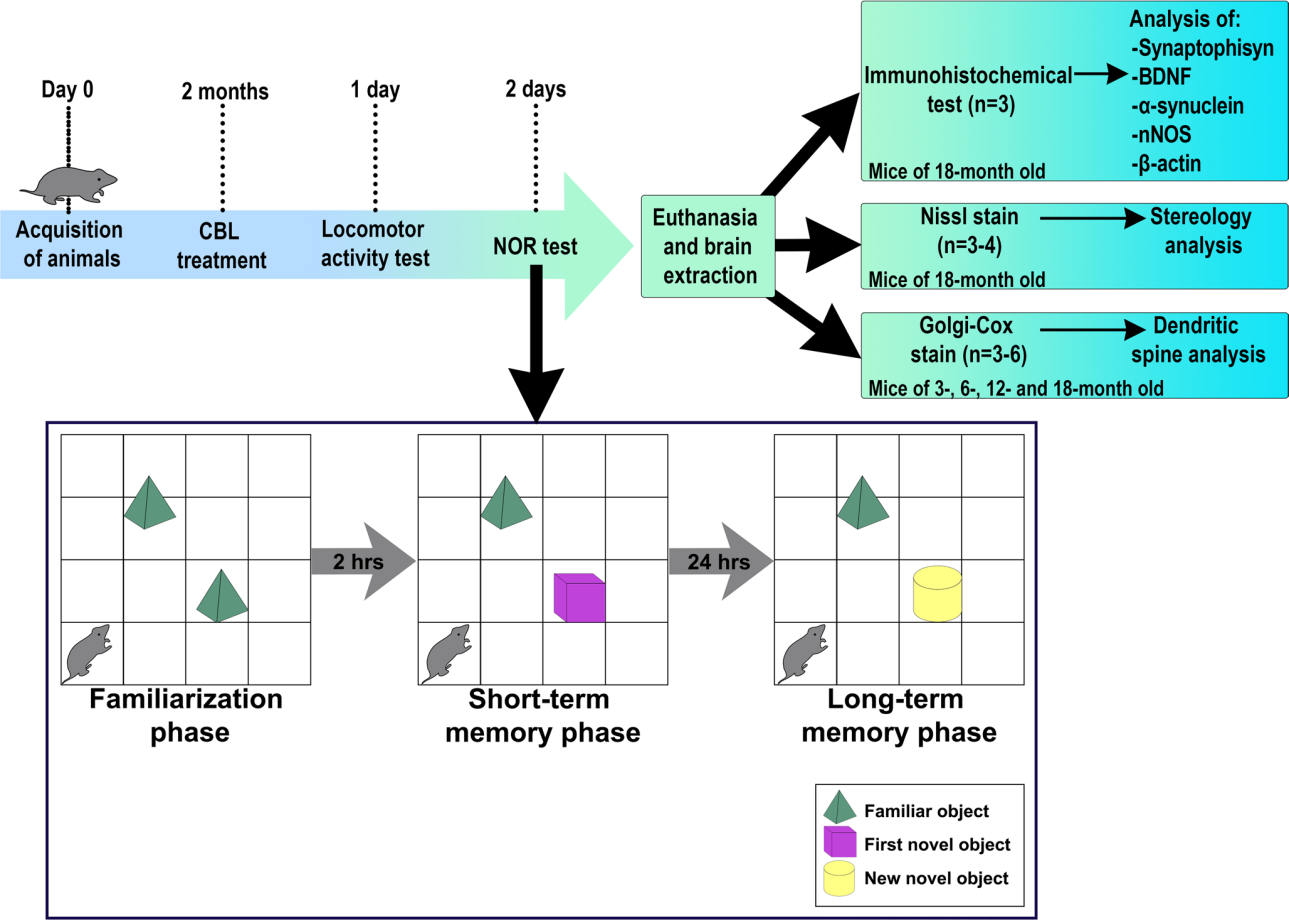
Methodology timeline and novel object recognition test. After acquiring the mice, the corresponding treatment was administered for two months. Behavioral testing was then conducted, beginning with locomotor activity the day after CBL treatment. Twenty-four hours later, the novel object recognition test was conducted, which consists of three phases. In the familiarization phase the mice innately interacted with two identical objects until they were familiarized with the objects. In short- and long-term memory phase the mice spent more time interacting with the novel object compared to the familiar object

To quantify recognition memory, the NOR test was applied using the same box used in locomotor activity. This test was performed 24 h after the locomotor activity and consisted of three phases [40] (Fig. 1). *Familiarization phase.* Two identical objects were placed in the test box, then the mouse was introduced and allowed to explore freely. *Short-term memory phase.* This phase was carried out 2 h after the familiarization phase. It consisted of replacing a familiar object with a novel object, then reintroducing the mouse for free exploration. The time spent with the familiar and novel objects were measured. *Long-term memory phase.* This phase was carried out 24 h after the short-term phase. Here, the novel object was replaced by a new novel object and the mouse was reintroduced to explore freely once again, and the time spent with the previously novel and the new novel object were recorded. Both the test box and the objects used were washed and disinfected between subjects. For analysis, the time each rodent spent exploring each of the objects was quantified, then the discrimination index (DI) was calculated using the following equation:

$$\:DI=\frac{N.\:O.}{N.\:O.\:+\:\:F.\:O.}$$

*N.O.* represents the time in seconds that the rodent spent exploring the novel object, and *F.O.* is the time spent exploring the familiar object. Id DI > 0.5 indicates that the animal spent more time exploring the novel object, and DI < 0.5 indicates that the rodent explored the familiar object longer.

The object interaction was quantified by counting the direct physical contacts with the objects. It was considered contact if the subject explored with olfactive senses and made vibrissae exploration with or without paw contact. Paw contact that was not accompanied by exploratory behavior was excluded (e.g., climb or lean onto the object). Moreover, only animals that spent ≥ 20 s exploring objects were considered for the analysis [41]. The score of each mouse was manually estimated in a double-blind manner by trained personnel.

#### Tissue Preparation

To quantify dendritic morphology, Golgi-cox staining [42] was used according to previous published methodology [43]. Mice were anesthetized using an overdose of sodium pentobarbital (75 mg/kg ip). Subsequently, a resection was performed to expose the heart to proceed with an intracardiac perfusion with physiological buffer. Immediately after, the brains were extracted and placed in Golgi-cox solution for 30 days for impregnation and fixation. After this time, 200 μm coronal sections of the brain were obtained using a vibratome (Leica) and sections were placed on gelatinized slides. The stain was then developed using sodium hydroxide for 30 min, the reaction was stopped with Kodak rapid fixer, and the tissues were dehydrated using alcohol and cleared with xylene. Finally, the tissues were covered with a coverslip and synthetic resin.

Observation of dendritic morphology was performed using an optical microscope coupled to a camera lucida (DMLS 2000, Leica Microscope) with a zoom of 1000X for dendritic spine density and 2000X for dendritic spine morphology. The regions analyzed were layer 3 and 5 of the PFC (Bregma 2.34 to −0.1 mm), hippocampal regions CA1, CA3, the dentate gyrus (DG) (Bregma − 1.46 to −2.54 mm) and basolateral amygdala (BLA) (Bregma − 0.58 to −2.06 mm). These regions were identified using a stereotaxic atlas [44].

#### Density and Morphology of Dendritic Spines

The number of dendritic spines was determined by drawing a 30 μm segment of a dendrite of 10 neurons from each region/animal (*n* = 5–6 in 3-, 6- and 12-month-old groups, *n* = 3 in 18-month-old group) [13]. Basal distal dendrites of pyramidal neurons of PFC, CA1, CA3, and BLA, and distal dendrites of granule neurons of the DG were analyzed. The number of spines was counted to determine the density of spines in 10 μm distance. The same dendrites were used to analyze dendritic morphology. For this purpose, the 100 most distal spines were classified according to their morphology into the following categories: *Thin (T)*, thin neck and small head; *Mushroom (M)*, short neck and broad; *Stubby (S)*, distinct neck/head; *Bifurcated (B)*, 2 heads; and *Unclassified (U)*, no resemblance to any of the other classifications. For pyramidal neurons (in PFC, CA1, CA3 and BLA) the basal dendrites were analyzed, for granular neurons (in DG) the dendrites analyzed were in the general dendritic arbor. In all cases, the dendrites analyzed were those that were most distal.

### Immunohistochemical Test

After mice were sacrificed as described above, they were perfused with paraformaldehyde 4% (PFA) and the brains (*n* = 3 for each group) were immediately fixed in PFA for 5–10 days and subsequently embedded in paraffin. Coronal sections of 10 μm were obtained using a microtome (Leica systems) and placed on slides. The samples were deparaffinized with xylene and then rehydrated with decreasing concentrations of ethanol and finally with water. Antigen retrieval was performed using citrate buffer (10mM, pH = 6) at 100 °C for 10 min. The non-specific staining was blocked using 2% immunoglobulin G (IgG) free albumin for 30 min. The cellular permeability to the antibody was increased using 0.2% Triton X-100 for 30 min. Slides were incubated for 24 h at 4 °C with the primary antibody to Syn (Cell Signaling #36406, Clone: D8F6H, 1:100), BDNF (Abcam, clone: 3C11, 5 µg/mL), α-synuclein (Cell Signaling #2647, clone: Syn204, 1:1000), nNOS (Cell Signaling #4231, Clone: C7D7, 1:200), and β-actin (Cell Signaling #3700, clone: 8H10D10, 1:3000). Afterward, slides were washed with phosphate buffered saline and incubated with the corresponding secondary antibody (Jackson Inmunoresearch Rhodamine Red™-X (RRX) anti-Rabbit for Syn; Jackson Inmunoresearch Rhodamine Red*™-X (RRX)* anti-Mouse for BDNF, α-synuclein, and β-actin; Cell Signaling anti-rabbit IgG Alexa Fluor^®^ 488 for nNOS) for 4 h at 4 °C. Finally, the slices were mounted with VectaShield-DAPI to stain the cellular nucleus (Vector Labs., CA, USA) and then observed in a fluorescence microscope (Leica DMLS). A total of 2 photomicrographs were taken by region of each slide (≈ 3 slices by region/animal) for each hemisphere given a total of 8–12 images of each region per brain. The images were obtained using the ImagePro Premier software at 40X and the analysis was performed using Image J software by counting reactive cells.

### Stereological Test

Neuronal density was determined using slides with paraffinized coronal samples of 30 μm size. Neuronal staining was performed via Nissl stain using cresyl violet as described in previous publications using *n* = 3–4 brains per group [43]. The observations were made in an optical microscope (Olympus BH2) using the Stereo Investigator System software (MicroBrightField, Williston) for cellular counting. For the analysis, two 5-µm thick guard zones were established above and below the measured zone in each section, thus the middle zone of 20-µm thickness in each section was analyzed. For the analysis of each sample, a Gundersen Error Coefficient (m = 1) < 0.05 was considered a reliable estimate.

### Statistical Analysis

All spine morphometry and IHC measures were averaged for each animal (mean of 10 neurons per brain in spines analysis, mean of 8–12 images per brain in IHC analysis). The obtained results are expressed as mean ± the standard deviation of the mean (SEM) and were analyzed using 95% of confidence intervals for all primary outcomes. The normality of the data was tested using the Shapiro-Wilk test. Behavioral and spine results were analyzed using a two-way ANOVA. The independent variables were treatment (VH or CBL) and age (3-, 6-, 12-, or 18-months-old). Inmunohistochemistry and stereology results were analyzed using t-tests. All the p-values were adjusted for multiple comparisons using the Two-stage linear step-up false discovery rate procedure of Benjamini, Krieger and Yekutieli (FDR-BKY), significant effects were considered for *q* < 0.05 (Q = 5%). For NOR results, a post hoc One-sample t-test (or Wilcoxon test) was performed comparing each group vs. 0.5 to confirm whether each group, on its own, showed significant memory. Those results with q < 0.05 were considered significant differences. The statistic partial eta-squared (η^2^_p_) was used as prospective power analysis to determine the strength of the effect considering η^2^_p_ > 0.14 as a large effect size for two-way ANOVA results. For the t-student results, we used Cohen’s D statistic adjusted for Hedges’ g (adjustment for small samples) to estimate the effect size, considering g > 0.8 as a large effect. The analysis was performed in GraphPad Prism software version 7.0 (California, USA). All behavioral assessments and immunological/histological analyses were performed by an experimenter blind to group allocation, with blinding maintained until all data were formally analyzed. Outcome measures included: behavioral performance assessed through standardized behavioral tests; neuronal morphology and density analyses; and molecular marker analyses, specifically Syn, BDNF, α-synuclein, nNOS, and β-actin.

## Results

### CBL Attenuated the Age-Induced Locomotor Activity and Recognition Memory Decline

**Fig. 2:**
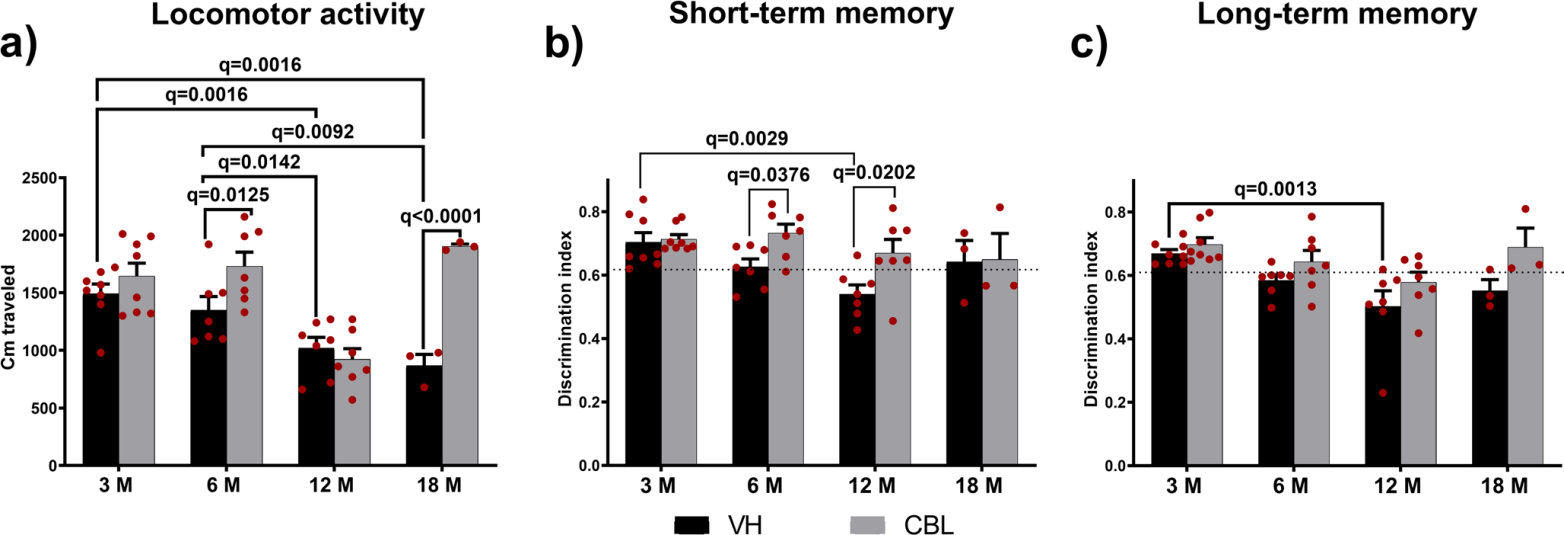
Changes in locomotor activity and recognition memory due to age and CBL treatment. **a** Locomotor activity. The 12- and 18-month-old mice have significantly less locomotion compared to 3- and 6-month-old mice. **b** Short-term recognition memory. Age causes a significant decline in discrimination index. CBL increased this parameter selectively in 18-month-old rodents. **c** Long-term recognition memory. There is a significant decline in recognition memory due to age

Locomotor activity was evaluated in the open field for 10 min (Fig. 2a). The results showed a reduction in the distance traveled in the 12- and 18-month-old mice compared to the 3- and 6-month-old mice (Two-way ANOVA, F(3, 2) = 14.96, *p* < 0.0001, η^2^_p_ = 0.516). CBL treatment produced a significant increase locomotor activity in 6- and 18-month-old mice (two-way ANOVA, F(1, 42) = 19.82, *p* < 0.0002, η^2^_p_ = 0.320).

Recognition memory was analyzed using the NOR test at two different times, short- and long-term. Analysis of short-term memory (Fig. 2b) showed a decrease in the DI of the 12-month-old rodents compared to those of 3 months (Two-way ANOVA, F(3, 42) = 4.053, *P* = 0.0128, η^2^_p_ = 0.224). CBL treatment produced a significant increase in recognition memory in 6- and 12-month-old rodents (Two-way ANOVA, F(1, 42) = 8.892, *P* = 0. 0196, η^2^_p_ = 0.123). The One Sample test showed a significantly higher DI in both the 3- and 6-month-old groups (p-values; 3 M-VH = 0.0078, 3 M-CBL = 0.0078, 6 M-VH = 0.0022, 6 M-CBL = 0.0002), and in the 12-month-old group treated with CBL(*P* = 0.0077). No significant differences were found in either of the 18-month-old groups. Analysis of long-term recognition memory (Fig. 2c) showed significant changes due to age (two-way ANOVA, F(3, 42) = 7.848, *p* = 0.0003, η^2^_p_ = 0.359). Post hoc analysis shows a significant reduction in long-term recognition memory in the 12-month group compared to the 3-month group. While CBL treatment induce an main effect (Two-way ANOVA, F(1, 42) = 9.284, *P* = 0.0040, η^2^_p_ = 0.181) but without significant changes between age groups. The One Sample test showed a similar pattern, i.e., significantly higher DI in both groups of 3- and 6-month-olds (p-values; 3 M-VH < 0.0001, 3 M-CBL = 0.0078, 6 M-VH = 0.0029, 6 M-CBL = 0.0069), and in the 12-month-old mice treated with CBL (0.0488). No significant differences were found in the control 12-month-old group or in any of the 18-month-old groups.

### CBL Attenuated Age-Induced Reductions in Spine Density

**Fig. 3:**
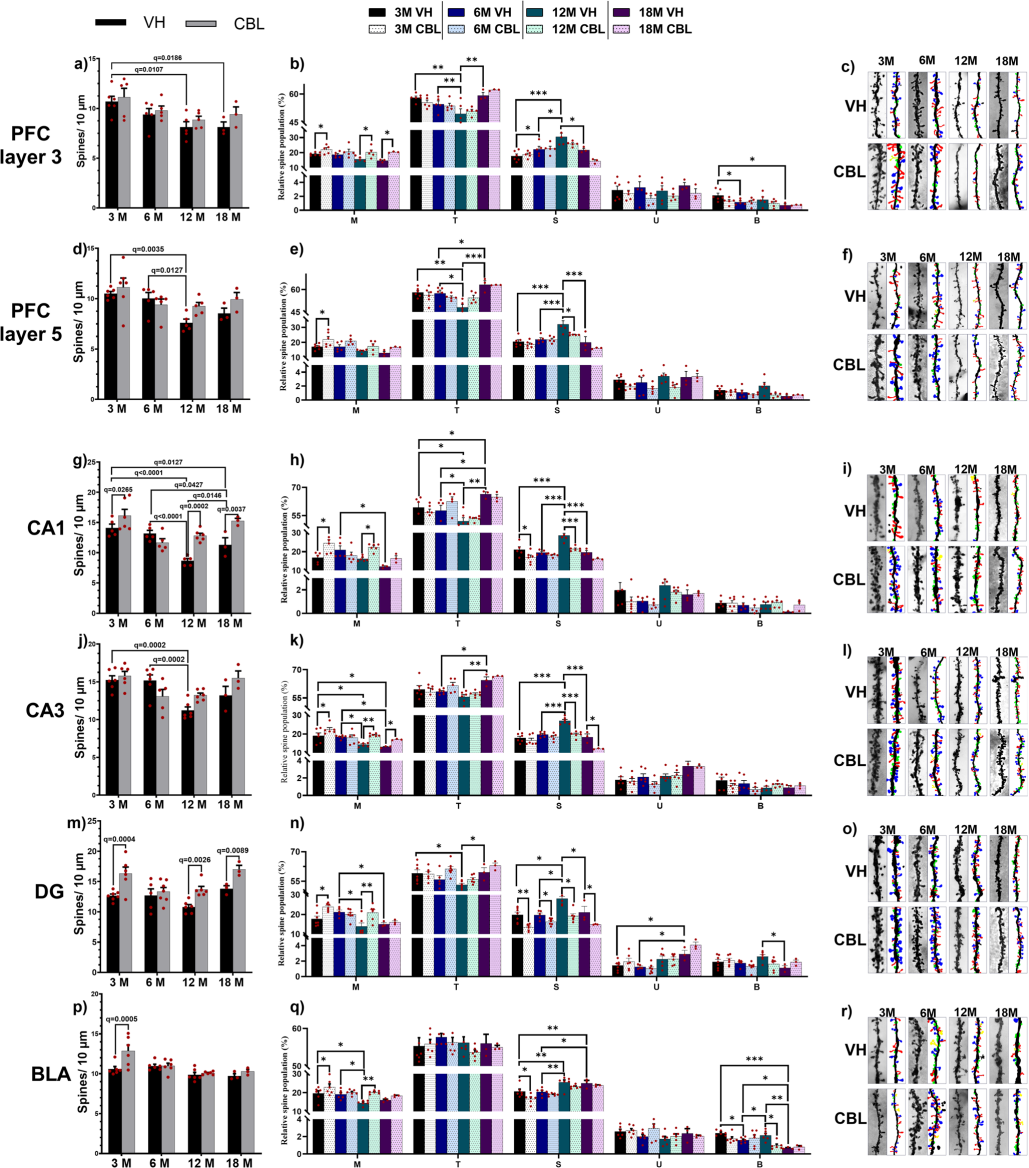
Dendritic spine density and morphology. **a** Dendritic spine density of the PFC layer 3. **b** Dendritic spine morphology of PFC layer 3. **c** Representative photography of distal dendrites by age and treatment in PFC layer 3. **d** Dendritic spine density in PFC layer 5. **e** Dendritic spine morphology of PFC layer 5. **f** Representative photography of distal dendrites by age and treatment of the PFC layer 5. **g** Dendritic spine density of the CA1. **h** Dendritic spine morphology of CA1. **i** Representative photography of distal dendrite by age and treatment of CA1. **j** Dendritic spine density of CA3. **k** Dendritic spine morphology of CA3. **l** Representative photography of distal dendrite by age and treatment of CA3. **m** Dendritic spine density of DG. **n** Dendritic spine morphology of DG. **o** Representative photography of distal dendrite by age and treatment in the DG. **p** Dendritic spine density of the BLA. **q** Dendritic spine morphology of the BLA. **r** Representative photography of distal dendrite by age and treatment in the BLA. **c**, **f**, **i**, **l**, **o**, **r** mushroom spines (blue), thin spines (red), stubby spines (green), bifurcated spines (yellow). Multiple comparison FDR-BKY *q < 0.05, **q < 0.001, ***q < 0.0001

Analysis of dendritic spines showed a decrease in the number of dendritic spines in pyramidal layer 3 (Fig. 3a) and 5 (Fig. 3d**)** neurons of the PFC. In PFC L3, the 12- and 18-month-old mice showed a significant reduction in dendritic spine density compared to the 3-month-old vehicle group (Two-way ANOVA, F(3, 29) = 7.085, *p* = 0.0010, η^2^_p_ = 0.422, Fig. 3a). In PFC L5, the 12-month-old CBL treated group showed a significant reduction in dendritic spine density compared to the 3- and 6-month-old vehicle groups (Two-way ANOVA, F(3, 30) = 6.448, *P* = 0.0017, η^2^_p_ = 0.392, Fig. 3d). In the 18-month-old CBL group, a non-significant reduction was observed compared to the 3- and 6-month-old vehicle groups (q = 0.0567, 0.1375, respectively). No age-induced decreases in dendritic spine density were observed in the CBL-treated groups.

In CA1 hippocampal pyramidal neurons, there was a decrease in dendritic spine density and a compensatory effect due to CBL (Fig. 3g and j). In the CA1 region, a significant decrease was observed in the 12- and 18-month-old groups compared to the 3- and 6-month-old rodents (two-way ANOVA, F(3, 30) = 14.95, *p* < 0.0001, η^2^_p_ = 0.599, Fig. 3g). Similar findings were obtained in the neurons of the CA3 region of the hippocampus with the 12-month-old group (Two-way ANOVA, F(3, 33) = 9.491, *p* = 0.0001, η^2^_p_ = 0.463, Fig. 3j). CBL treatment produced a significant increase in the dendritic spine density in the CA1 region in the 3-, 12- and 18-month-old groups compared to their control groups (two-way ANOVA, F(1, 30) = 17.44, *p* = 0.0002, η^2^_p_ = 0.367, Fig. 3g). No significant changes in the CA3 region were observed with CBL treatment.

Finally, age did not significantly affect the dendritic spine density in the granular neurons of the DG (Fig. 3m) or in the pyramidal neurons of the BLA (Fig. 3p). CBL treatment produced a significant increase the spine density in the 3-, 12- and 18-month-old groups compared to their control groups in the DG (two-way ANOVA, F(1, 35) = 23.46, *p* < 0.0001, η^2^_p_ = 0.401, Fig. 3m). In the BLA, CBL treatment only produced a significant increase in spine density in 3-month-old mice (two-way ANOVA, F(1, 34) = 6.524, *p* = 0.0153, η^2^_p_ = 0.161, Fig. 3p).

### CBL Reduced Age-Induced Alterations in the Proportion of Mushroom and Stubby Spines

#### Prefrontal Cortex

The analysis of the pyramidal neurons in PFC L3 (Fig. 3b) showed that mushroom spines do not suffer a significant decrease due to age (two-way ANOVA, F(3, 28) = 2.856, *P* = 0.0550, η^2^_p_ = 0.234), while a trend towards decrease is observed mainly in 12- and 18-month-old mice (q = 0.0774). However, treatment with CBL produced a significant increase in mushroom spines in 3-, 12-, and 18-month-old mice compared to their control groups (two-way ANOVA, F(1, 28) = 18.61, *p* = 0.0002, η^2^_p_ = 0.399). In thin spines, a significant reduction was observed in 12-month-old mice compared to 3- and 6-month-old mice, and an increase in 18-month-old mice compared to the 12-month-old group (two-way ANOVA, F(3, 28) = 12.02, *p* < 0.0001, η^2^_p_ = 0.562). CBL treatment did significantly change thin spines in either group. On the other hand, a significant increase in stubby spines was observed in 12-month-old mice compared to 3, 6 and 18-month-old mice (two-way ANOVA, F(3, 28) = 17.42, *p* < 0.0001, η^2^_p_ = 0.651). CBL treatment does not produce significant changes in stubby spines; however, their levels showed a trend toward restoration. No unclassified spine changes are observed either due to age or CBL treatment. A significant decrease in bifurcated spines was observed in 6- and 18-month-old mice compared to the 3-month-old group (Two-way ANOVA, F(3, 28) = 3.236, *P* = 0.0371, η^2^_p_ = 0.257). CBL treatment did not produce significant changes in bifurcated spines in either group.

In the pyramidal neurons of PFC L5 (Fig. 3e), a trend towards a decrease in mushroom spines due to age was found (two-way ANOVA, F(3, 29) = 4.575, *p* = 0.0096, η^2^_p_ = 0.321); however, no significant changes were found in the post-hoc test. CBL treatment increased mushroom spines only in 3-month-old mice (two-way ANOVA, F(1, 29) = 13.41, *p* = 0.0010, η^2^_p_ = 0.316). A significant increase in mushroom spines was observed in the 18-month-old group compared to the 6-month-old mice, as well as the observation of a decrease in thin spines in 12-month-old mice compared to the 3-, 6-, and 18-month-old groups (Two-way ANOVA, F(3, 29) = 9.738, *p* = 0.0001, η^2^_p_ = 0.501). Treatment with CBL did not produce significant changes in thin spines. In the stubby spines of this region, a significant increase was observed in the 12-month-old rodents compared to the 3-, 6-, and 18-month-old groups (two-way ANOVA, F(3, 29) = 17.83, *p* < 0.0001, η^2^_p_ = 0.648). CBL treatment significantly reduced stubby spines in 12-month-old mice compared to their control group (two-way ANOVA, F(1, 29) = 7.665, *p* = 00.97, η^2^_p_ = 0.209). No significant differences were observed in unclassified spines by either age or treatment. In bifurcated spines, the ANOVA test found no significant changes due to age (Two-way ANOVA, F(3, 29) = 2.397, *p* = 0.0885, η^2^_p_ = 0.198), or to the treatment (Two-way ANOVA, F(1, 29) = 3.880, *p* = 0.0585, η^2^_p_ = 0.118).

#### Hippocampus

Analysis of dendritic spine types in CA1 pyramidal neurons (Fig. 3h) showed a significant age-related reduction in mushroom spines in 18-month-old mice compared to 6-month-old mice two-way ANOVA, F(3, 30) = 4.086, *p* = 0.0152, η^2^_p_ = 0.290). Meanwhile, CBL treatment significantly increased mushroom spines in the 3- and 12-month-old groups compared to their control groups (two-way ANOVA, F(1, 30) = 10.80, *p* = 0.0026, η^2^_p_ = 0.264). No changes in thin spine density were observed with CBL treatment; however, a significant increase was found in 18-month-old mice compared to 12-month-old mice (Two-way ANOVA, F(3, 30) = 9.955, *P* = 0.0001, η^2^_p_ = 0.498). In stubby spines, the analysis revealed a significant increase due to age in 12-month-old mice compared to 3-, 6-, and 18-month-old mice (two-way ANOVA, F(3, 30) = 21.03, *p* < 0.0001, η^2^_p_ = 0.677). Treatment with CBL significantly reduced stubby spines in the 3- and 12-month-old groups compared to their control groups (two-way ANOVA, F(1, 30) = 26.73, *p* < 0.0001, η^2^_p_ = 0.471). No changes in unclassified or bifurcated spines were observed in this hippocampal region due to age or CBL treatment.

In the hippocampus CA3 region (Fig. 3k), the analysis showed a significant reduction of mushroom spines due to age in 12- and 18-month-old mice compared to 3- and 6-month-old mice (two-way ANOVA, F(3, 32) = 9.161, *p* = 0.0002, η^2^_p_ = 0.462). CBL treatment appeared reduced these reductions, even showing a significant increase in 3-, 12-, and 18-month-old mice compared to their control groups (two-way ANOVA, F(1, 32) = 15.06, *p* = 0.0005, η^2^_p_ = 0.319). Thin spine density was increased in 18-month-old mice compared to 6- and 12-month-old mice (two-way ANOVA, F(3, 32) = 8.308, *p* = 0.0003, η^2^_p_ = 0.347). No significant changes were observed due to CBL treatment. In stubby spines, a significant age-related increase was observed in 12-month-old mice compared to 3-, 6-, and 18-month-old mice (two-way ANOVA, F(3, 32) = 26.55, *p* < 0.0001, η^2^_p_ = 0.713). CBL treatment significantly attenuated this increase in 12- and 18-month-old mice compared to their control groups (two-way ANOVA, F(1, 32) = 32.89, *p* < 0.0001, η^2^_p_ = 0.506). Finally, in the unclassified and bifurcated spines, no changes associated with either age or treatment were observed.

#### Dentate Gyrus

In the DG (Fig. 3n), mushroom spine density was significantly decreased in 12- and 18-month-old mice compared to 6-month-old mice (two-way ANOVA, F(3, 34) = 5.056, *p* = 0.0053, η^2^_p_ = 0.308). CBL treatment significantly increased these spines in 3- and 12-month-old mice compared to their control groups (two-way ANOVA, F(1, 34) = 8.905, *p* = 0.0052, η^2^_p_ = 0.207). In thin spines, a reduction was observed in 12-month-old mice compared to 3- and 18-month-old mice (two-way ANOVA, F(3, 34) = 5.420, *p* = 0.0037, η^2^_p_ = 0.827); however, CBL treatment had no effect. A significant increase in stubby spines was observed in 12-month-old mice compared to 3-, 6-, and 18-month-old mice (two-way ANOVA, F(3, 34) = 14.30, *p* < 0.0001, η^2^_p_ = 0.557). CBL treatment significantly reduced stubby spines in all groups compared to their control groups (two-way ANOVA, F(1, 34) = 42.99, *p* < 0.0001, η^2^_p_ = 0.558). In addition, an increase in unclassified spines was found in 18-month-old mice compared to the 3- and 6-month-old groups (two-way ANOVA, F(3, 34) = 12.08, *p* < 0.0001, η^2^_p_ = 0.516) with no changes from CBL treatment. Finally, an increase in bifurcated spines was found in the 12-month-old group compared to the 18-month-old group (two-way ANOVA, F(3, 34) = 2.216, *p* = 0.1041, η^2^_p_ = 0.163), but CBL treatment did not have any effect.

#### Basolateral Amygdala

Analysis in the BLA (Fig. 3q) showed a decrease in mushroom spine density in 12-month-old mice compared to 3- and 6-month-old mice (two-way ANOVA, F(3, 33) = 6.207, *p* = 0.0018, η^2^_p_ = 0.360). CBL treatment significantly increased this spine density in 3- and 12-month-old mice compared to their control groups (two-way ANOVA, F(1, 33) = 13.77, *p* = 0.0008, η^2^_p_ = 0.294). A significant increase in stubby spines was observed in 12- and 18-month-old mice compared to the 3- and 6-month-old groups (two-way ANOVA, F(3, 33) = 17.79, *p* < 0.0001, η^2^_p_ = 0.617). CBL treatment delayed or attenuated these increases, even in the 3-month-old mice compared to their control group (two-way ANOVA, F(1, 33) = 8.595, *p* = 0.0061, η^2^_p_ = 0.206). 18-month-old mice exhibited a significant decrease in bifurcated spines compared to the 3-, 6-, and 12-month-old groups (two-way ANOVA, F(3, 33) = 8.209, *p* = 0.0003, η^2^_p_ = 0.427). The 6-month-old group also showed decreased bifurcated spine density compared with the 3- and 12-month-old groups. CBL treatment produced a significant decrease in bifurcated spine density inn12-month-old mice compared to their control group (two-way ANOVA, F(1, 33) = 4.635, *p* = 0.0387); however, the effect size (η^2^_p_ = 0.123) was not large. Neither aging nor CBL treatment changed the density of thin or unclassified spines.

### CBL Produced an Increase in Syn, BDNF and β-Actin in Aged Rodents

**Fig. 4:**
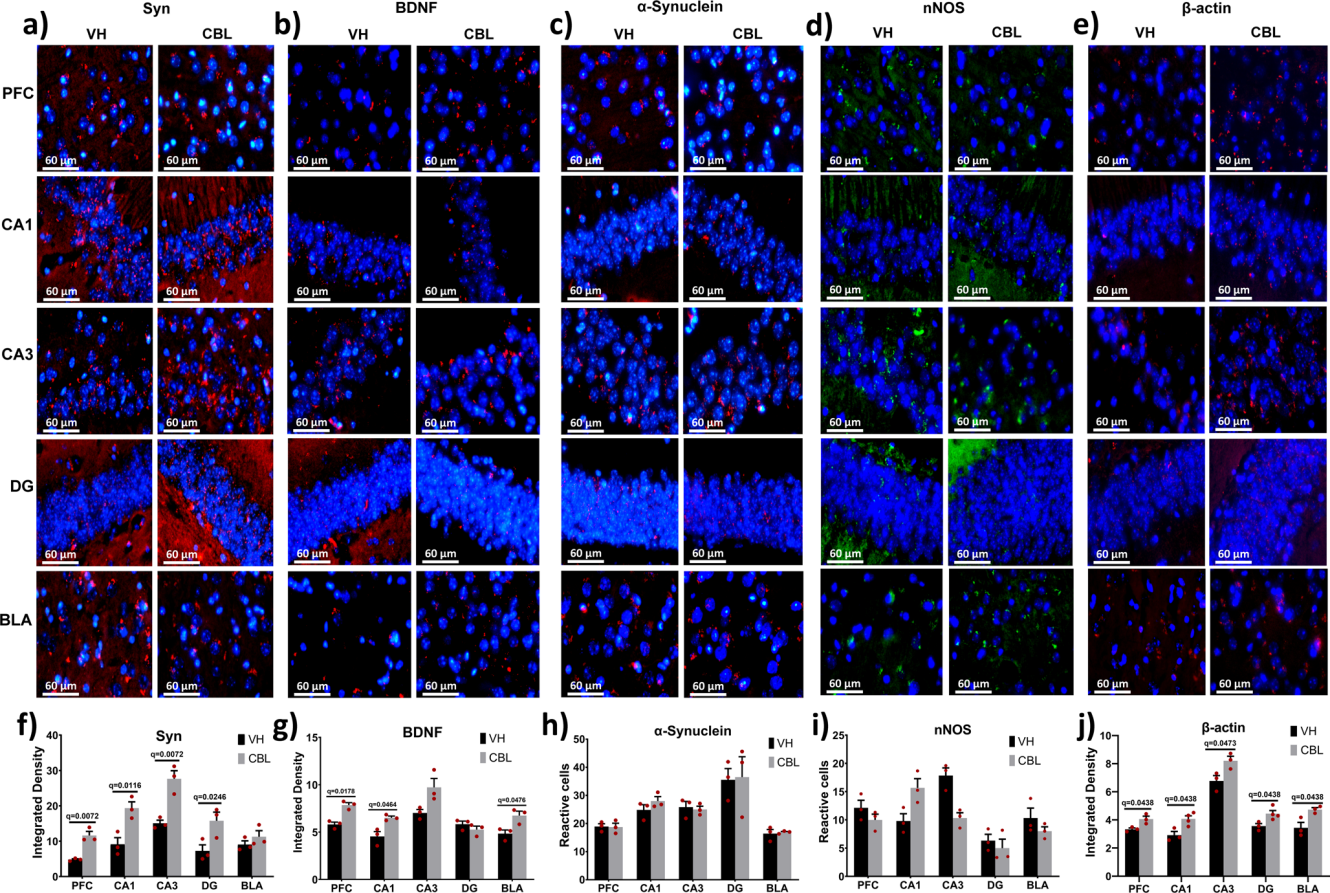
Results of immunofluorescence assays. **a** Immunofluorescence photographs of synaptophysin (red) and the cell nucleus (blue). **b** Immunofluorescence photographs of Brain Derived Neurotrophic Factor (red) and the cell nucleus (blue). **c** Immunofluorescence photographs of α-synuclein (red) and the cell nucleus (blue). **d** Immunofluorescence photographs of neuronal Nitric Oxide Synthase (green) and the cell nucleus (blue). **e** Immunofluorescence photographs of β-actin (red) and the cell nucleus (blue). **f** CBL increases synaptophysin in prefrontal cortex and hippocampus. **g** CBL increases the presence of Brain Derived Neurotrophic Factor in the prefrontal cortex, CA1 hippocampus, and basolateral amygdala. **h** α-synuclein is not affected by CBL treatment. **i** CBL treatment did not affect nNOS density. **j** CBL induces an increase in β-actin in each analyzed region. (PFC: Prefrontal cortex; DG: Dentate gyrus; BLA: basolateral amygdala; Syn; Synaptophysin; BDNF; Brain derived neurotrophic factor; nNOS: neuronal oxide nitric synthase)

Immunohistochemistry performed for the detection of Syn (synaptic marker), BDNF (neurotrophic factor), α-synuclein (indicator of pathological protein deposits), nNOS (marker of oxidative stress) and β-actin (cytoskeleton) in 18-month-old mice (Fig. 4). The results showed that CBL significantly increased synaptophysin in the PFC (FDR-BKY; q = 0.007275, g = 3.819), hippocampus CA1 (FDR-BKY; q = 0.0161610, g = 2.590), CA3 (FDR-BKY; q = 0.007275, g = 3.337) and DG (FDR-BKY; q = 0.024637, g = 1.853) (Fig. 4a and f). Regarding BDNF, an CBL induced increase was observed in the PFC (FDR-BKY; q = 0.01738, g = 3.945), hippocampus CA1 (FDR-BKY; q = 0.0466488, g = 1.898), and BLA (FDR-BKY; q = 0.047625, g = 2.211) (Fig. 4b and g). No changes in α-synuclein (Fig. 4c and h) or nNOS (Fig. 4d and i) were observed due to treatment. Finally, β-actin analysis revealed a significant increase in all the regions analyzed (FDR-BKY; PFC, CA1, DG, BLA q = 0.043803, CA3 = 0.047356, g = 2.080) in CBL treated groups (Fig. 4e and j).

### CBL Did not Alter Neuronal Density in Aged Mice

**Fig. 5:**
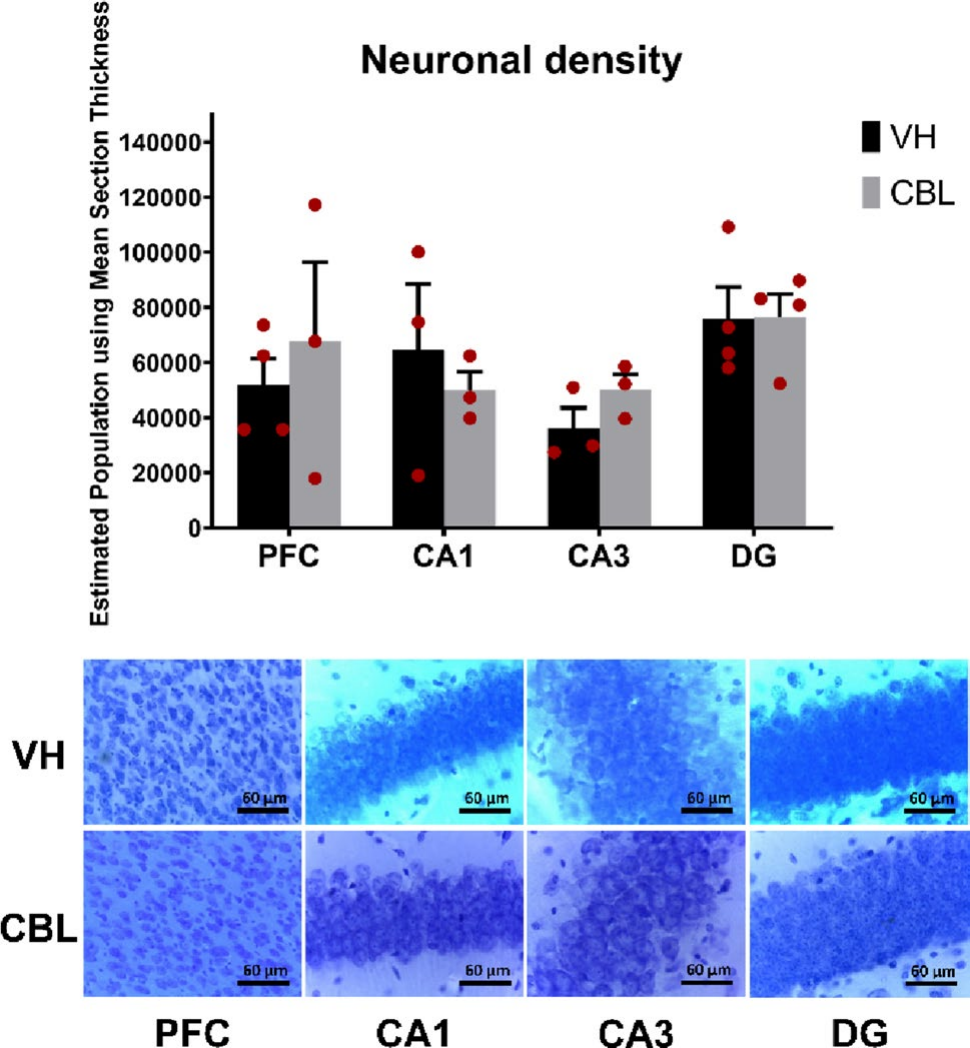
CBL effect on neuronal density. **a** Stereological analysis shows no significant changes from CBL treatment in any of the regions analyzed in aged rodents. **b** Representative photographs of each region analyzed

The estimation of neuronal density was carried out using the stereology technique. All samples obtained a Gundersen error coefficient (m = 1) between 0.02 and 0.05 (only one brain from the vehicle group of CA1 obtained 0.06). Analysis of the results did not show significant changes induced by CBL in any region (Fig. 5a). A non-significant increase in the PFC (t-test, *p* = 0.5286, g = 0.384) and CA3 (t-test, *p* = 0.2079, g = 0.074) was observed following CBL treatment.

## Discussion

In this study, we investigated the impact of aging on dendritic spines and recognition memory, and assessed whether CBL treatment could reduce age-induced alterations in these parameters. Additionally, we examined the effects of CBL on key molecular markers related to synaptic plasticity, oxidative stress and inflammation in aged mice. Aging significantly reduced locomotor activity in 12- and 18–month-old mice. It also induced deficits in short-term memory in the 12-month-old group compared to the 3-month-old group and in long-term memory relative to young controls. CBL selectively increased locomotion in aged mice and improved short-term memory in the 12-month-old group. Furthermore, aging reduced spine density in the PFC and CA1/CA3 subregions of the dorsal hippocampus, whereas CBL increased spine density in the BLA and CA1/DG. While aging reduced the proportion of thin and mushroom spines, CBL increased mushroom spine number in a region- and age-dependent manner. Importantly, CBL elevated β-actin, Syn and BDNF expression in most evaluated regions of aged mice. Taken together, these findings indicate that CBL confers structural, behavioral and molecular benefits that counteract age-related synaptic and cognitive decline.

### CBL Modifies the Aging Induced Reductions in Locomotor Activity and Memory Impairment Seen at Specific Ages

In agreement with previous studies in aged rodents, our study found that aging was associated with reduced locomotor activity [45]. The decrease in motor activity could be related to an age-related reduction in dopaminergic transmission related to motor regulation and exploratory motivation [46, 47]. Moreover, a correlation has been proposed between motor alterations of age and a reduction in dopamine levels [48], part of this age-related decline in motor function could be caused by typical aging-associated muscle [49], joint [50] and even visual system [51]. On the other hand, when analyzing CBL treatment effects, we observed increased locomotor activity in 18-month-old mice, but not in the rest of the groups. Similarly, other studies have found that CBL produces an increase in locomotor activity in aged rodents [52, 53] and juvenile rodents in an animal model of schizophrenia [54].

Recognition memory, which arises from the innate behavior of rodents to explore stimuli that are novel and non-aversive, was evaluated in this test. Rodents tend to spend more time exploring novel stimuli compared to familiar stimuli [55]. Therefore, this test assesses the memory of mice to distinguish familiar objects from those with which they have not interacted [41]. Alterations in this type of memory have been described in aging, neurodegenerative disorders, and substance abuse [45, 56, 57]. Our results show an age-dependent decline in recognition memory performance, as younger groups (3- and 6-month-old) exhibited significantly higher DI compared with a hypothetical value (0.5), while older groups (12- and 18-month-old) showed no significant values. These findings suggest that this type of memory is preserved during early adulthood but declines progressively. Interestingly, CBL treated mice displayed a higher DI than control groups mainly in the 3-, 6-, and 12-month-old groups, suggesting that this drug could exert a protective effect on this parameter. Other studies have shown a decrease in memory and spatial learning related to aging [45, 58]. Consequently, we evaluated whether CBL could reverse this decline, and we observed that the treatment significantly increased short-term memory in the 12-month-old mice. In the other age groups, and in long-term memory, only a non-significant CBL induced increase was observed. Similar results have been found evaluating spatial memory in aged rodents [59–61], in animal models with ethanol consumption [62], and in animal models of diabetes [63]. Furthermore, CBL has shown potential as a preventive therapy against memory decline in animal models of post-traumatic stress [64] and sleep deprivation [61]. Thus, this drug is considered as a possible treatment to improve cognitive abilities in aging [65].

### Region- and Age-Dependent Effects of Aging and CBL on Spine Density and Morphology

The present study quantified the density and morphology of dendritic spines in cortical and limbic regions at different ages. Dendritic spines are small extensions of dendrites that mainly correspond to the postsynaptic element of glutamatergic synapses. These structures show a high capacity for activity-dependent plasticity, which gives them the ability to adapt their morphology in response to stimulation, thus modifying the effectiveness of their synaptic transmission [66, 67]. The relevance of these plastic structures in memory and learning processes has been proposed, where the participation of LTP and LTD has been mentioned as processes that modify the shape and efficiency of dendritic spines [66, 68, 69]. However, the plasticity of these structures can also be modified by changes in the extracellular and intracellular environment, mainly due to the establishment of oxidative and inflammatory stress states [70], and the availability of neurotrophic factors [7], phenomena that are normally observed during aging. Our results show an age-induced reduction in the density of dendritic spines in the PFC and hippocampus, with no changes observed in the DG and BLA. Previous studies from our laboratory [13, 18, 71] and other groups [72] have shown reduced dendritic spine density with age in rodents. On the other hand, studies in human samples have also found a decrease in the density of dendritic spines and changes in the proportion of mushroom and stubby spines in pyramidal neurons of the orbitofrontal cortex associated with aging [73]. We found that CBL increased the number of dendritic spines in the CA1 and DG in aged rodents. Although the increase observed in the PFC did not reach statistical significance, it mitigated the significant reduction observed relative to the youngest group. These data show that CBL exerts its effect via structural changes in dendrites, thus reducing age-related dendritic atrophy. Other studies in animal models of schizophrenia [54], diabetes/hypertension [30–32], and aging [74] have also shown that CBL increases dendritic spines.

To the best of our knowledge, this is the first study where CBL show modifications in the type of spines. An increase in the density of dendritic spines may not be directly related to an improvement in synaptic transmission because the effectiveness of each dendritic spine is determined by its morphology [6, 67]. The dendritic spines with the best synaptic transmission are the mushroom type, as they have a larger area of ​​synaptic contact and arise from LTP processes. On the other hand, those spines with less efficient synaptic transmission are the stubby type with a lower synaptic contact area and derive from LTD processes [75, 76]. Thin spines are considered transitional spines that will later formalize into mushroom or stubby spines, depending on the stimulation they receive and the cellular environment [77, 78], while bifurcated spines appear to be participating in the formation of new dendritic spines [79–81]. Therefore, an increase in dendritic spine density could be a compensatory mechanism but may not result in an improvement in synaptic transmission. To investigate this phenomenon, the proportion of different dendritic spine types were evaluated. Our results show a gradual age-induced reduction in the number of mushroom spines, as well as an increase in stubby spines. When treatment with CBL was evaluated, a reduction in age-related alterations in these dendritic spines was observed, mainly in the PFC and hippocampus. Taken together, the above results suggest that part of the causes of age-related cognitive decline could be related to changes in the density and morphology of dendritic spines, mainly in regions related to memory and learning processes [82, 83]. Likewise, CBL treatment could reduce these changes, thus observing improvements in the performance of recognition memory tests.

### CBL Treatment Increases Several Factors That Shape the Type of Dendritic Spines in Aged Mice

The effectiveness of dendritic spines depends on stimulation and the milieu of the environment. Therefore, a key part of our work was undertaken to determine whether the structural modifications in spines by CBL was derived in part from changes in the intra- and extra-cellular environment. For this, 18-month-old mice were used to quantify Syn, BDNF, nNOS, and β-actin as biomarkers of synaptic plasticity, and α-synuclein as a marker of protein aggregates associated with Parkinson’s disease.

Syn is a protein present in axon terminals where it participates in the release of neurotransmitters, therefore its presence can be used as a synapse marker. Syn belongs to the family of integral proteins of the MARVEL domain [86] and participates in the fusion and recycling of synaptic vesicles, and in conjunction with the SNARE machinery, specifically participating in the formation of the pore fusion complex that regulates the exocytosis of synaptic vesicles [87]. Therefore, a correlation is observed between attenuated synaptic transmission and a reduction in the presence of synaptophysin [88]. Our results show that CBL increases Syn levels in the PFC and all subregions evaluated within the hippocampus. Studies have showed reductions in presynaptic proteins, including Syn, in in aged rodents [89–91], and that CBL increases this protein in the hippocampus, DG and neocortex [74, 84, 92–94]. An increase in Syn in limbic-cortical regions is associated with better performance in learning and memory, even in aging [8, 95–98]. However, studies with an animal model of diabetes have reported that CBL did not change the amount of Syn levels in the cerebellum [99] [89–91]. Therefore, our results suggest that CBL may mitigate the age-associated decline in this protein.

BDNF is a neurotrophic factor that exerts its effects by interacting with its specific Tyrosine kinase B receptors (TrkB) and the consequent activation of intracellular signaling pathways that culminate in the activation of processes that improve synaptic transmission, neurogenesis, learning and memory [100]. Part of the function of BDNF is the regulation of excitatory and inhibitory synapses at the pre- and post-synaptic level in both LTP and LTD, even promoting protein synthesis [101]. This neurotrophin seems to be essential for the molecular mechanisms of spine morphogenesis, especially in the hippocampus and neocortex [102]. The intracellular mechanisms that trigger the effects of BDNF occur from the activation of the PI3K/Akt/CREB, ERK/MAPK and PLC/PKC signaling pathways [103, 104]. Furthermore, BDNF could in turn directly regulate the expression of genes or activation of other signaling pathways after its dendritic release, its internalization by the postsynaptic neuron and its subsequent retrograde transport [105–107]. In addition, it has been suggested that BDNF deficits are related to the pathogenesis of several neuronal disorders such as Parkinson’s, Huntington’s, Alzheimer’s, depression, chronic stress and anxiety [108–113]. Based on this information, knowing whether CBL affects BDNF levels would indicate whether this drug is acting on signaling pathways that promote neuronal plasticity. In our project, the expression of BDNF increased significantly in the hippocampus and PFC of rodents treated with CBL. Our results resemble those found by Hernández-Hernández (2018), who reported an increase in BDNF in senile rats after treatment with Neuropeptide-12, a CBL derivative [74]. Likewise, other studies have seen that CBL increases the amount of BDNF in vitro [74, 114], in animal models of epilepsy [115], and depression [116, 117] and in the serum of human patients with Alzheimer’s disease [118]. However, other studies in aged rats treated with CBL did not find changes in BDNF but observed alterations in the amount of neurotrophin receptors [59], which is related to studies that show an age-induced decrease in the expression of TrkB but not in the presence of BDNF in humans [119]. Nonetheless, other studies have reported reductions in BDNF levels in aged rats compared to young counterparts [120]. Therefore, the improvements found due to CBL could also be a consequence of an increase in BDNF and its innate neurotrophic activity.

Actin microfilaments participate in the formation and maintenance of the structure of dendritic spines [121–123]. Moreover, studies in aged rodents show a decrease in the amount of actin in brain tissue [124]. Therefore, a novel aspect of this study is that, to our knowledge, no previous research has evaluated the effect of CBL on β-actin levels in the brain. Our results show that CBL treatment increases β-actin in the PFC, hippocampus and BLA in aged rodents. This may be due to the fact that one of the components of CBL are actin fragments [125], which could support the maintenance of the cytoskeleton. The dynamics of β-actin allow the morphological change of dendritic spines in response to stimulation for their differentiation into mushroom spines [126–128]. Alterations in actin filaments have been proposed to contribute to cognitive decline during aging [129]. Treatment with CBL seems to reduce this atrophy in the cytoskeleton of aged mice, promoting better dynamics in the plasticity of dendritic spines. However, it is important to acknowledge that β-actin immunoreactivity does not necessarily reflect functional or structural cytoskeletal integrity [130]. Variations in β-actin IHC signal may arise from technical factors such as section thickness, antigen retrieval, or antibody penetration, rather than true biological differences. Therefore, the interpretation of increased β-actin staining should be made with caution and require incorporation of more specific cytoskeletal markers, such as phalloidin for F-actin, to confirm these observations.

nNOS is an enzyme with regulated expression in dendritic spines in response to synaptic stimulation [131, 132]. Its function is the production of nitric oxide (NO) for the regulation of synaptic plasticity, neurogenesis, memory and learning [133]. Our data show that CBL increases nNOS levels in the CA1 region of the hippocampus while decreasing them in the CA3 region. Alteration in nNOS expression has been linked to mental and neurodegenerative disorders [134]. Excessive production of NO can lead to events such as excitotoxicity, oxidative stress and cell death [135], and is associated with disorders such as depression [136, 137], Parkinson’s [138] and Alzheimer’s disease [139, 140]. NO participates as a retrograde messenger, inducing glutamate release in the axon terminal during LTP processes. Therefore, excess NO produces an exacerbated release of glutamate leading to excitotoxicity due to a massive entry of calcium, activating cell death pathways [141, 142]. To verify CBL’s effects on nNOS, it is important to first determine whether nNOS levels vary as a function of age. It has been seen that CBL attenuate increased expression of nNOS, which occurs in Parkinson’s disease [143], Alzheimer’s disease [144], and diabetes and hypertension [145]. Likewise, CBL treatment decreases NO levels in the PFC and hippocampus in animal models of hypertension [85]. Altogether, it seems that nNOS expression varies in a region-specific matter.

The peak of expression of α-synuclein in the brain occurs during postnatal development in rodents and humans and is particularly important for synaptogenesis [146]. After mid-adulthood, expression of the protein gradually declines but pathological accumulation can occur during aging or neurodegenerative disease [147]. As such, the presence of α-synuclein, the main protein of Lewy bodies and a marker of Parkinson’s disease, was analyzed [148, 149]. The rationale is that since its important in synaptogenesis, perhaps abnormally high levels could lead to cognitive impairment [150]. Synucleinopathies are neurodegenerative diseases characterized by the presence of pathological brain inclusions composed of α-synuclein filaments, such as Parkinson’s disease and Lewy body dementia [150]. In neurons, α- and β-synucleins are mainly located in presynaptic terminals, close to synaptic vesicles [151]. In our study, no significant differences in were observed in α-synuclein immunoreactivity in any of the regions analyzed. It has been observed that CBL can reduce α-synuclein levels in animal models of Parkinson’s disease which present significant increases in this protein, but not in control groups [143]. Therefore, the action of CBL might not affect this protein because it has been reported that its expression in mice decreases with age in regions such as the substantia nigra [152]. Recently, it has been proposed that the pathological effects of α-synuclein are expressed only in cases of accelerated aging, and not in normal aging phenotypes [48].

### CBL Does not Modulate Neuronal Density in the Regions Analyzed in Aged Mice

In our study, CBL treatment did not show significant changes in neuronal density in 18-month-old mice, only a trend towards an increase in the PFC and CA3 subregion of the hippocampus was observed. It has been proposed that CBL selectively attenuates neuronal density in models where it is reduced; however, no significant changes are observed in rodents with a normal neuronal density, therefore promoting survival [28]. Studies in rats of different strains have shown that there are no changes in neuronal density due to age in regions of the brain stem or sensory cortex [153]. Another study showed a decrease in neuronal density in regions such as the lateral geniculate nucleus [154]. Despite this, the changes observed could coincide, if the trend continues, with other studies that have shown that CBL increases the neuronal population in the nucleus accumbens and PFC in animal models of schizophrenia [28, 54]. Moreover, CBL could increase the survival of stem cells in animal models of Alzheimer’s disease [155]. One mechanism by which CBL could maintain neuronal density is by reducing the rate of apoptosis, which has been seen in animal models of traumatic brain injury [156], vascular dementia [157], cerebral ischemia and hemorrhage [158, 159]. Likewise, it has been shown that it can increase neurogenesis in rat models of moderate closed head injury [160, 161], stroke [162], Alzheimer’s disease [163], and traumatic brain injury [164]. These results suggest that CBL may exert region-specific neuroprotective actions that preserve neuronal populations under conditions of vulnerability. Continued investigations with extended treatment windows will be necessary to determine whether these preliminary indications translate into robust increases in neuronal density.

### CBL Increases Various Plasticity-Related Molecules Important in Dendritic Spine Maintenance

The mechanisms of CBL action are pleiotropic because its composition consists of a set of diverse molecules. In this study we observed that CBL treatment increased spine density and mushroom spines along with increases in BDNF, Syn and β-actin, all important in spine structure. Spines are the site where most synapses occur and are classified among different subtypes with functional properties [6]. Among those, mushroom spines can be stable over months and are considered as functional spines connected to an axonal bouton [165]. On the other side, thin spines could transition into stable mushroom spines and acquire a functional synapse [166]. Dendritic spine enlargement and stabilization represent key structural correlates of synaptic plasticity [167]. Spine enlargement is a rapid, activity-dependent process driven by actin cytoskeleton remodeling, primarily through ß-actin polymerization, which increases spine head volume and synaptic efficacy [168]. However, for these transient changes to persist, neurotrophic support, particularly through BDNF, is essential to promote actin reorganization, local protein synthesis, and long-term structural stabilization [169]. In parallel, presynaptic adaptations such as increased Syn expression reflect enhanced vesicle recycling and synaptic maturation, indicating coordinated pre- and post-synaptic remodeling [170]. Together, the interplay between ß-actin-mediated cytoskeletal dynamics, BDNF-dependent trophic signaling, and Syn-associated presynaptic adjustments underlie the consolidation of stable spines induced by CBL that support enduring forms of learning and memory.

A limitation of the present study is the exclusive use of male rats, which prevents assessment of potential sex-specific differences in the parameters measured across developmental stages. Considering the well-documented sex-dependent trajectories in brain maturation and plasticity, the omission of female subjects restricts the generalizability of the findings. Reasons for this limitation at the present time are that fluctuations in ovarian hormones influence dendritic spine density and behavior introducing intrinsic variability [171, 172]. Moreover, pharmaceuticals may exhibit differential metabolism, receptor expression, and efficacy between males and females [173]. Finally, age-related neuronal and cognitive decline proceeds differently in males and females, meaning that sex differences may change direction or magnitude across the lifespan [174]. Therefore, future studies should incorporate both sexes to address these differences and enhance translational relevance.

In conclusion, aging is accompanied by morphological and physiological alterations at the level of synaptic connections in regions involved in cognitive processes such as the hippocampus and the PFC. These alterations are characterized by a decrease in spine density and changes in spine morphology with possible implications in synaptic function, ultimately impairing performance in recognition memory. CBL treatment shows beneficial effects by maintaining dendrites, suggesting its potential as a prophylactic and adjunctive treatment to mitigate age-related cognitive decline.

## Data Availability

Data is available upon reasonable request.
